# COVID-19 Vaccination-Related Delayed Adverse Events among Patients with Systemic Lupus Erythematosus

**DOI:** 10.3390/jcm12247542

**Published:** 2023-12-07

**Authors:** Mrinalini Dey, Bohdana Doskaliuk, Julius Lindblom, Elena Nikiphorou, Chris Wincup, Madiha Fathima, Sreoshy Saha, Syahrul Sazliyana Shaharir, Wanruchada Katchamart, Phonpen Akarawatcharangura Goo, Lisa Traboco, Yi-Ming Chen, Esha Kadam, James B. Lilleker, Arvind Nune, John D. Pauling, Vishwesh Agarwal, Dzifa Dey, Carlos Enrique Toro Gutierrez, Carlo Vinicio Caballero, Hector Chinoy, COVAD Study Group, Rohit Aggarwal, Vikas Agarwal, Latika Gupta, Ioannis Parodis

**Affiliations:** 1 Centre for Rheumatic Diseases, King’s College London, Weston Education Centre, Cutcombe Road, London SE5 9RJ, UK; mrinalini.dey@nhs.net (M.D.); enikiphorou@gmail.com (E.N.); 2 Department of Pathophysiology, Ivano-Frankivsk National Medical University, 76018 Ivano-Frankivsk, Ukraine; doskaliuk_bo@ifnmu.edu.ua; 3 Division of Rheumatology, Department of Medicine Solna, Karolinska Institutet and Karolinska University Hospital, 17176 Stockholm, Sweden; julius.lindblom@ki.se (J.L.); madiha.fathima2417@gmail.com (M.F.); 4 Rheumatology Department, King’s College Hospital, London SE5 9RS, UK; c.wincup@ucl.ac.uk; 5 Mymensingh Medical College, Mymensingh 2200, Bangladesh; sreoshysaha11@gmail.com; 6 Faculty of Medicine, Universiti Kebangsaan Malaysia, Cheras, Kuala Lumpur 56000, Malaysia; ssazliyana@gmail.com; 7 Division of Rheumatology, Department of Medicine, Faculty of Medicine Siriraj Hospital, Mahidol University, Bangkok 10100, Thailand; wanda.katchamart@gmail.com (W.K.); covadstudy@gmail.com (COVAD Study Group); 8 Department of Medicine, Queen Savang Vadhana Memorial Hospital, Chonburi 20000, Thailand; phonpen@msn.com; 9 Department of Medicine, Section of Rheumatology, St. Luke’s Medical Center-Global City, Taguig 1630, Philippines; 10 Division of Allergy, Immunology and Rheumatology, Department of Internal Medicine, Taichung Veterans General Hospital, Taichung 402, Taiwan; blacklark@gmail.com; 11 Department of Medical Research, Taichung Veterans General Hospital, Taichung 402, Taiwan; 12 Seth Gordhandhas Sunderdas Medical College and King Edwards Memorial Hospital, Mumbai 400001, Maharashtra, India; kadamesha13@gmail.com; 13 Division of Musculoskeletal and Dermatological Sciences, Faculty of Biology, Medicine and Health, Manchester Academic Health Science Centre, The University of Manchester, Manchester M13 9PL, UK; james.lilleker@manchester.ac.uk (J.B.L.); hector.chinoy@nhs.net (H.C.); drlatikagupta@gmail.com (L.G.); 14 Manchester Centre for Clinical Neurosciences, Salford Royal NHS Foundation Trust, Salford M16 0TT, UK; 15 Southport and Ormskirk Hospital NHS Trust, Southport PR8 6PN, UK; arvind.nune@nhs.net; 16 Bristol Medical School Translational Health Sciences, University of Bristol, Bristol BS8 1QU, UK; johnpauling@nhs.net; 17 Department of Rheumatology, North Bristol NHS Trust, Bristol BS9 4RJ, UK; 18 Mahatma Gandhi Mission Medical College, Navi Mumbai 401208, Maharashtra, India; vishwesh002@gmail.com; 19 Rheumatology Unit, Department of Medicine and Therapeutics, University of Ghana Medical School, College of Health Sciences, Korle-Bu, Accra KB 460, Ghana; drdzifadey@gmail.com; 20 Reference Center for Osteoporosis, Rheumatology and Dermatology, Pontifica Universidad Javeriana Cali, Cali 760046, Colombia; carlostororeumatologo@gmail.com; 21 Department of Medicine, Hospital Universidad del Norte, Barranquilla 081001, Colombia; carvica@gmail.com; 22 Department of Rheumatology, Salford Royal Hospital, Northern Care Alliance NHS Foundation Trust, Salford M6 8HD, UK; 23 Division of Rheumatology and Clinical Immunology, University of Pittsburgh School of Medicine, Pittsburgh, PA 15261, USA; aggarwalr@upmc.edu; 24 Department of Clinical Immunology and Rheumatology, Sanjay Gandhi Postgraduate Institute of Medical Sciences, Lucknow 226014, Uttar Pradesh, India; vikasagr@yahoo.com; 25 Department of Rheumatology, Royal Wolverhampton Hospitals NHS Trust, Wolverhampton WV10 0QP, UK; 26 Department of Rheumatology, City Hospital, Sandwell and West Birmingham Hospitals NHS Trust, Birmingham B71 4HJ, UK; 27 Department of Rheumatology, Faculty of Medicine and Health, Örebro University, 70281 Örebro, Sweden

**Keywords:** systemic lupus erythematosus, COVID-19, vaccines, delayed adverse events

## Abstract

Background: The safety profile of COVID-19 vaccination is well documented, but hesitancy among people with immune-mediated inflammatory diseases, often immunocompromised, remains high, partially due to a scarcity of data on safety over a longer term. We herein aimed to assess delayed adverse events (DAEs) occurring >7 days after COVID-19 vaccination in systemic lupus erythematosus (SLE) versus other rheumatic autoimmune diseases (rAIDs), non-rheumatic AIDs (nrAIDs), and healthy controls (HCs). Methods: Self-reported data were captured within the COVID-19 Vaccination in Autoimmune Diseases (COVAD)-2 online survey, which comprised >150 centres and responses from 106 countries, between February and June 2022. Logistic regression analysis adjusting for important confounders (age, sex, ethnicity) was used to compare groups. Results: Of 7203 eligible individuals, 882 (12.2%) patients had SLE, 3161 (43.9%) patients had rAIDs, 426 (5.9%) patients had nrAIDs, and 2734 (38.0%) were HCs. SLE patients had a median age of 39 years (IQR: 31–50); 93.7% were women. SLE patients reported, more frequently, major DAEs (OR: 1.6; 95% CI: 1.2–2.0; *p* = 0.001) and hospitalisation (OR: 2.2; 95% CI: 1.4–3.4; *p* < 0.001) compared to HCs, severe rashes (OR: 2.4; 95% CI: 1.3–4.2; *p* = 0.004) compared to people with rAIDS, and hospitalisation (OR: 2.3; 95% CI: 1.1–4.9; *p* = 0.029) as well as several minor DAEs compared to people with nrAIDs. Differences were observed between vaccines in terms of frequency of major DAEs and hospitalisations, with the latter seen more frequently in patients receiving the Moderna vaccine. People with SLE with no autoimmune multimorbidity less frequently reported overall minor DAEs compared to SLE patients with comorbid nrAIDs (OR: 0.5; 95% CI: 0.3–1.0; *p* = 0.036). Conclusion: Hospitalisations post-vaccination were more frequent in SLE patients than in HCs. Monitoring of SLE patients following COVID-19 vaccination can help in identifying DAEs early, informing patients about expected DAEs, and supporting patients, especially those with autoimmune multimorbidity.

## 1. Introduction

Vaccination against Coronavirus Disease 2019 (COVID-19) has significantly reduced the severity and mortality resulting from the pandemic, and is known to be safe in healthy populations [1]. Increasing evidence suggests that vaccination against COVID-19 is also safe and effective in those who were excluded from trials, for example those with immune-mediated inflammatory diseases (IMIDs), who are at increased risk of complications from COVID-19 [2,3]. Vaccine hesitancy remains common in the general population and especially among people with IMIDs, for example, due to fear of disease flares and side-effects on a background of symptoms secondary to their condition as well as their comorbidities [4,5,6]. Increasing evidence suggests a positive short-term vaccination safety and adverse event (AE) profile in this high-risk group [7,8,9,10].

Fewer data exist on delayed AEs (DAEs), i.e., those that occur >7 days post-vaccination, especially in people with IMIDs, who are often immunocompromised [11]. Recent work within the COVID-19 Vaccination in Autoimmune Diseases (COVAD) study has demonstrated a low incidence of DAEs in people with idiopathic inflammatory myositis (IIM), while ongoing work within the COVAD study in people with rheumatoid arthritis (RA) suggests similar reassuring outcomes [12].

Data from the COVAD collaboration has previously shown that up to 80% of people with systemic lupus erythematosus (SLE) reported AEs within seven days following COVID-19 vaccination, but these were mostly minor and comparable to AE frequencies reported by healthy individuals [9]. Data from pregnant and breastfeeding women with SLE receiving the COVID-19 vaccine have also been reassuring, including an absence of pregnancy complications [13]. People with SLE are among those most at risk of complications from severe respiratory infections, including COVID-19, due to multiple factors including a dysregulated immune system, multimorbidity—especially in patients with nephritis and pulmonary involvement—and often high levels of immunosuppression [3,14,15]. AEs and tolerability following COVID-19 vaccination at greater than seven days post-vaccination are hitherto unexplored in people with SLE.

We aimed to investigate DAEs, reported longer than seven days post-vaccination, in patients with SLE compared with patients with rheumatic autoimmune diseases other than SLE (rAIDs), patients with non-rheumatic autoimmune diseases (nrAIRDs), and healthy controls (HCs) within the COVAD-2 study. 

## 2. Materials and Methods

### 2.1. Study Design

This study was conducted as a part of the COVAD-2 initiative i.e., a cross-sectional, multi-centre, patient self-reported online survey. The protocol for the study has been previously reported [11]. Participants were informed and consented electronically. Ethics approval was obtained from the Sanjay Gandhi Postgraduate Institute of Medical Sciences (SGPGIMS) ethics committee (IEC Code: 2021-143-IP-EXP-39). The study adhered to the Checklist for Reporting Results of Internet E-Surveys (CHERRIES) [16].

### 2.2. Data Collection

The questionnaire was comprehensively tested before release, revised, translated into 18 languages by a team of experts, and subsequently disseminated via www.surveymonkey.com (accessed on 3 November 2023). It was circulated extensively by the COVAD study group across >150 centres worldwide, patient support groups, and social media platforms between February and June 2022.

Data on multiple factors were collected, including demographics, diagnosis of SLE or other autoimmune diseases, comorbidities, concomitant treatment, current status of disease, COVID-19 infection history, and outcomes, such as hospitalisation and the need for oxygen therapy. COVID-19 vaccination details were recorded, as well as early-onset (≤7 days) or delayed-onset (>7 days) post-vaccination AEs in accordance with the Center for Disease Control and Prevention [CDC] criteria. Rheumatic and non-rheumatic autoimmune diseases are listed in Supplementary Table S1, and adverse events are listed in Supplementary Table S2. Patient-reported outcomes were collected using the Patient Reported Outcomes Measurement Information System (PROMIS) [17]. The study included individuals over the age of 18 years, including those with multiple overlapping autoimmune diseases.

### 2.3. Data Extraction

Data were extracted on 10 July 2022. Participants were included if they completed the entire survey and had received at least one dose of the COVID-19 vaccine at the time of completion. Relevant outcome measures, delayed-onset self-reported vaccine AEs, sociodemographic and clinical characteristics, and vaccination status were among the variables extracted for analysis.

### 2.4. Active versus Inactive SLE

Active and inactive SLE disease activity four weeks prior to vaccination were assessed by the patient’s response to the question “What was the status of your autoimmune disease in the four weeks prior to the first dose of COVID-19 vaccine?” Patients who reported active or worsening/static/improving disease were categorised as having “active disease”, while those who reported “inactive” disease comprised the inactive group. For patients who responded with “I don’t know” or “Other”, disease status was assessed on an individual basis using follow-up questions about their symptoms, joint swelling, and immunosuppressant medication use in the six months prior to vaccination. 

### 2.5. Adverse Events Post-Vaccination

DAEs were classed as major or minor, as detailed in Supplementary Table S2 [18]. Survey participants reported DAEs among a prespecified list but were able to report additional DAEs not listed among those prespecified options as “others” via an open-ended question. 

### 2.6. Statistical Analysis

Data distribution was determined using Kolmogorov–Smirnov and Shapiro Wilk tests. Descriptive statistics for continuous variables are presented as the median and interquartile range (IQR). To assess the difference between categorical variables, the chi-squared (*χ*^2^) test was used, and for continuous variables, the Mann–Whitney *U* test was used. Fisher’s exact test was employed to compare categorical data in case of counts of less than 5 in at least one cell upon tabulation. 

Comparisons of DAEs were conducted between individuals with SLE and individuals with rAIDs, individuals with nrAIRDs, and HCs. Subgroup analyses were performed based on the type of vaccine received, SLE activity, autoimmune and non-autoimmune comorbidities, and immunosuppressive therapy. Subgroup analysis was also performed for SLE patients with versus without mental health comorbidities, recorded in the questionnaire as anxiety, bipolar disorder, depression, eating disorders, insomnia, schizophrenia, and substance use disorders. The multivariable binary logistic regression models adjusted for factors defined a priori, i.e., age, sex, and ethnicity. When analysing by drug, since hydroxychloroquine (HCQ) is considered background therapy for all patients with SLE, we also accounted for other concomitant immunosuppressive medications in the model of HCQ. In the multivariable models, statistical significance was set at *p* < 0.05. Results are reported as odds ratios (ORs) with 95% confidence intervals (CIs). Statistical analyses were performed using SPSS Statistics version 26 (IBM Corp., Armonk, NY, USA).

## 3. Results

### 3.1. Baseline Characteristics

A total of 10,783 individuals across 106 countries responded to the survey, of whom 7203 had received at least one vaccine dose and had provided complete responses and hence formed the study cohort (Table 1). The included respondents had an overall median age of 44 years (IQR 34, 56), with the majority being female (75.0%) and Caucasian (42.2%). The study cohort comprised 882 (12.2%) patients with SLE, 3161 (43.9%) patients with rAIDs, 426 (5.9%) patients with nrAIDs, and 2734 (38.0%) HCs. People with SLE were on average younger than the rest of the cohort, with a median age of 39 years (IQR: 31–50), with the majority being women (93.7%).

### 3.2. Post-COVID-19 Vaccination-Associated DAEs in People with SLE, Compared to Other rAIDs, nrAIDs, and HCs

Among patients with SLE, 155 (17.6%) respondents reported minor DAEs, while major DAEs were reported by 118 individuals (13.4%) (Table 2). When compared to HCs, people with SLE reported major DAEs significantly more frequently in the multivariable analysis (OR: 1.6; 95% CI: 1.2–2.0; *p* = 0.001). Several minor DAEs were also reported more frequently in the SLE group, including rash (OR: 3.4; 95% CI: 1.7–6.6; *p* < 0.001), visual disturbances (OR: 3.5; 95% CI: 1.7–7.2; *p* = 0.001), bleeding and/or bruising (OR: 3.9; 95% CI: 1.6–9.5; *p* = 0.003), and petechial rash (OR: 4.2; 95% CI: 1.1–15.5; *p* = 0.030) (Table 2). With regard to major DAEs, people with SLE reported marked difficulty in breathing (OR: 2.0; 95% CI: 1.1–3.5; *p* = 0.020) and severe rash (OR: 2.2; 95% CI: 1.2–4.1; *p* = 0.011) more frequently than HCs, as well as hospitalisation (OR: 2.2; 95% CI: 1.4–3.4; *p* < 0.001).

Compared to people with rAIDS, SLE patients more frequently reported severe rashes in the multivariable analyses (OR: 2.4; 95% CI: 1.3–4.2; *p* = 0.004; Table 3). In comparison to people with nrAIDs, people with SLE more frequently reported several minor DAEs, including body ache (OR: 2.1; 95% CI: 1.2–3.7; *p* = 0.008), joint pain (OR: 2.4; 95% CI: 1.3–4.3; *p* = 0.005), and nausea and/or vomiting (OR: 3.0; 95% CI: 1.2–7.3; *p* = 0.017; Table 4). Reporting of major DAEs was comparable between people with SLE and nrAIDs; however, people with SLE more frequently reported hospitalisation (OR: 2.3; 95% CI: 1.1–4.9; *p* = 0.029).

### 3.3. Post-COVID-19 Vaccination-Associated DAEs across Vaccine Types

Among people with SLE, 558 (63.3%) had received the BNT162b2 (Pfizer) vaccine, 288 (32.7%) received ChadOx1 nCOV-19 (Oxford/AstraZeneca), 161 (18.3%) received mRNA-1273 (Moderna), and 73 (8.3%) received Sinovac-CoronaVac. It should be noted that some patients had received more than one dose with more than one type of vaccine at the time of completing the survey, accounting for these data.

In the multivariable binary logistic regression analysis, people with SLE who had received ChadOx1 nCOV-19 (Oxford/AstraZeneca) reported diarrhoea to a significantly greater extent compared to those having received other vaccines (OR 2.6; 95% CI: 1.1–6.2; *p* = 0.037; Supplementary Table S3). People with SLE who had received the Moderna vaccine reported overall major DAEs (OR 1.7; 95% CI: 1.1–2.8; *p* = 0.022) as well as hospitalisation (OR 3.0; 95% CI: 1.6–5.6; *p* < 0.001) more frequently compared to those receiving other vaccines. 

### 3.4. Post-COVID-19 Vaccination-Associated DAEs and Type of Immunosuppression

Drug treatments with conventional synthetic immunosuppressants were recorded in the SLE cohort; those included methotrexate (MTX; 13.2%; n = 116), mycophenolate mofetil (MMF; 19.6%; n = 173), azathioprine (AZA; 20.2%; n = 178), and HCQ (69.6%; n = 614). A greater proportion of patients with SLE who were on MTX reported difficulty in breathing (OR: 3.3; 95% CI: 1.3–8.3; *p* = 0.012) or swelling of the extremities (OR: 5.8; 95% CI: 1.9–17.6; *p* = 0.002) compared to SLE patients who were not on MTX (Supplementary Table S4). Patients with SLE receiving MMF less frequently reported fever compared to SLE patients who were not on MMF (OR: 0.4; 95% CI: 0.2–0.9; *p* = 0.036). Patients with SLE taking HCQ less frequently reported overall minor DAEs but did report hospitalisation to a greater extent compared to SLE patients who were not on HCQ (OR: 2.9; 95% CI: 1.3–6.6; *p* = 0.010). However, these associations abated after adjustment for other concomitant medications (OR: 0.5; 95% CI: 0.1–1.7; *p* = 0.272).

Post-COVID-19 vaccination-associated DAEs in people with active and inactive SLE

In univariable logistic regression analysis, people with active SLE more frequently reported nausea and vomiting (OR: 2.3; 95% CI: 1.1–5.1; *p* = 0.033). However, reports of both minor and major DAEs were comparable between people with active and inactive SLE in the multivariable models. 

### 3.5. Post-COVID-19 Vaccination-Associated DAEs in People with Only SLE, SLE and rAID Comorbidity, SLE with nrAID Comorbidity and Active vs. Inactive SLE

In multivariable logistic regression analysis, people with SLE but no autoimmune multimorbidity less frequently reported dizziness (OR: 0.5; 95% CI: 0.2–1.0; *p* = 0.039) and bleeding and/or bruising (OR: 0.2; 95% CI: 0.1–0.8; *p* = 0.017) compared to SLE patients with comorbid rAIDs. Reports of major AEs were comparable between the two groups.

Compared to people with SLE and nrAID comorbidities, people with SLE but no autoimmune multimorbidity overall reported minor AEs less frequently (OR: 0.5; 95% CI: 0.3–1.0; *p* = 0.036), while reports of major AEs were comparable between the two groups.

People with SLE and mental health disorders more frequently reported headache (OR: 2.2; 95% CI: 1.3–3.6; *p* = 0.002), severe rash (OR: 3.2; 95% CI: 1.3–7.5; *p* = 0.008), and anaphylaxis (OR: 7.7; 95% CI: 1.5–38.8; *p* = 0.013). 

With regard to disease activity, people with self-reported active SLE overall reported similar frequencies of DAEs compared to individuals with inactive disease.

## 4. Discussion

Our study has provided data on delayed-onset AEs >7 days post-vaccination in people with SLE receiving the COVID-19 vaccine. To our knowledge, this was the first study to examine delayed-onset AEs following COVID-19 vaccination in this high-risk multimorbid population, including comparisons with people with other rAID and nrAID diagnoses. We have demonstrated overall reassuring results with mainly minor AEs, which were self-limiting. However, patients with SLE more frequently reported hospitalisations compared with healthy individuals and patients with non-rheumatic autoimmune conditions, a finding that should not be overlooked. 

Vaccine hesitancy remains high among people with SLE, and has persisted during the COVID-19 pandemic and vaccination strategies within the community [19]. Driving factors for this include concerns about potential long-term vaccine AEs, which are further exacerbated by a lack of safety and tolerability data from large-scale prospective studies conducted in this patient population and deliberate exclusion from vaccine trials. We have previously demonstrated reassuring early-onset AE data in people with SLE [9], which are now largely reinforced by the data on delayed-onset AEs presented herein.

Our data are consistent with previous works from the COVAD collaboration, which demonstrated a good AE profile at >7 days post-vaccination in people with IIMs [12]. They also align with findings in large registry studies, including the EULAR Coronavirus Vaccine (COVAX) study, in which one-third of patients experienced AEs at <7 days post-vaccination (clinician-reported), although most were minor and/or self-limiting [20]. In a single-centre study comprising 466 people with SLE in the USA, 74% of the patients self-reported minor AEs within 7 days of COVID-19 vaccination [21]. Similarly, the international VACOLUP study, also comprising patient-reported data via a web-based survey, demonstrated that 45% of patients with SLE experienced AEs after the first vaccine dose, and 53% after the second, of a total of 696 people with SLE [22]. Crucially, however, those AEs were not found to impair functional ability. Importantly, this study also explored flares of SLE post-vaccination and noted that flares and vaccine AEs may sometimes be indistinguishable.

Our data yielded some interesting results, not reported previously to this degree of granularity. Overall, people with SLE reported minor AEs more frequently compared to HCs. However, minor AEs yielded the greatest differences; examples of such minor AEs are rash and bruising. Major AEs reported more frequently in the SLE group included severe rash and hospitalisation. Concerns have previously been raised regarding SLE flares following vaccination, although there is little evidence to support this [21,23]. Multiple cases of SLE flares following vaccination against COVID-19 have been reported in the literature, and while there is no confirmed association between the two, there may be a benefit in monitoring people with SLE post-vaccination to help identify DAEs. Data from the Global Rheumatology Alliance, which recorded clinician-reported symptoms following COVID-19 vaccination in people with rAIDs, reported that flares were uncommon [24]. While our study focused on DAEs and did not aim to report signs or symptoms of SLE flare post-vaccination, it is important to keep in mind that many DAEs may mimic a flare and vice versa, posing challenges in distinguishing across reasons for hospitalisation.

In the SLE population, in whom antiphospholipid syndrome is highly prevalent, it is also relevant to consider the previous, albeit later proven unfounded, concerns regarding the association between the Oxford–AstraZeneca vaccine and an increased risk of clotting events [25]. While clotting events were not specifically explored in our study, related AEs, such as the swelling of limbs, were. Within the survey, the phrase “swelling in the extremities” described swelling at any site in the limbs (whole or part). Importantly, the Joint Council for Vaccination and Immunisation in the UK declared in 2022 that there were no safety concerns with regard to this vaccine and clotting events, and that it should be continued to be offered to all patients as the benefits greatly outweighed the risks [26]. Reassuringly, in our study, people with SLE who had received the Oxford–AstraZeneca vaccine reported diarrhoea more frequently that SLE patients who had received other vaccines against COVID-19, but no increased frequencies of symptoms such as limb swelling or breathing difficulties were noted.

It is interesting to note the greater reported frequency of hospitalisation following COVID-19 vaccination in people with SLE compared to both HCs and those with nrAIDs. Unfortunately, our survey did not record the exact cause of hospitalisation, but given the relative multimorbidity of this patient cohort, a greater underlying degree of chronic inflammation, and vulnerability to infections, there are several factors that may account for this finding, and caution should be exercised in its interpretation. The increased risk of infections is of particular relevance, as this patient group would likely have been prioritised for vaccination in several countries when levels of COVID-19 infection in the community were still high. Unfortunately, with the limited patient-reported data available to us, it remained beyond the scope of this paper to assess, in depth, certain risk factors for critical disease in the context of infection and vaccination.

We recorded the use of conventional immunosuppressants, with people taking MTX more frequently reporting difficulty in breathing and the swelling of the extremities in our cohort. However, the underlying explanation of this association remains unclear. This result should also be interpreted with caution given the relatively small sample size. It is worth noting that people with SLE receiving MMF, a drug usually given in patients with more moderate/severe disease, especially in the presence of lupus nephritis [27], in fact had relatively fewer reported or comparable frequencies of AEs with those on other or on no immunosuppressants. Again, the sample size of people receiving MMF was relatively small within our cohort. It is also worth mentioning that the decreased frequency of reported fever may have been due to better disease control and therefore fewer episodes of fever linked to disease flare, which also highlights the challenge of distinguishing between adverse events and symptoms stemming from active SLE. Nonetheless, this finding is reassuring. A possible explanation might be that patients withheld their treatment in the 2–4 weeks prior to and/or after their vaccination, as suggested by multiple national and international guidelines [28,29,30] for boosting the immune response to vaccination [31,32]. However, consideration has to be given to the potential propensity to flare during that period, and therefore, the consequences of holding immunosuppressants remain unclear with regard to delayed-onset AEs. 

We also generated data for people with SLE and comorbidities. People with SLE who had comorbid rAIDs or nrAIDs reported more frequent minor AEs than those with SLE alone, with multiple AEs in those with nrAIDs, and especially regarding dizziness and bleeding and/or bruising for those with rAIDs. Partially, an explanation for this could be the increased comorbidity and therefore symptom burden experienced by these patients, which in turn may have contributed to non-specific symptoms. The presence of coexisting autoimmune diseases also suggests a propensity to an increased underlying state of inflammation compared to patients with SLE alone. People with SLE and mental health disorders more frequently reported headaches and severe rashes. Mental health disorders may be related or unrelated to a patient’s SLE, which often complicates the attribution of symptoms to the disease. Headache may also be attributed to SLE; approximately 20–55% of people with SLE experience migraine-like headaches during their disease course, depending on the cohort under study [33]. Related symptoms of fatigue, anxiety, and depression are highly prevalent in people with SLE and likely contributed to this finding in our cohort [34,35]. It is worth noting that people with SLE and mental health disorders reported a greater frequency of anaphylaxis compared to those without them. However, the explanation for this is unclear and the results should be treated with caution, especially given the solely patient-reported information and potential loss of meaning when the survey was translated to multiple languages. 

Our study has some limitations, a major one being recall bias owing to data deriving from self-reports. Moreover, the internet-based survey may have excluded people with poor internet connectivity, low socioeconomic status, and impaired access to technology, who may also be more vulnerable to AEs. Patients were asked about AE symptoms at >7 days post-vaccination, but not specifically regarding symptoms of SLE flare, which may have been overlapping, making it difficult to determine whether the reported symptoms had occurred due to vaccination, flare, or another factor such as concurrent infection. However, it should be noted that flares of SLE following vaccination, and humoral vaccine responses, have been well documented elsewhere and were not within the scope of this study. While it would have been useful to identify clusters of DAEs by region, no obvious patterns were noted, and the DAEs were in fact very heterogeneous with regard to organ system or body region affected. This study may be regarded as a case–control study (with cases being those with SLE, compared to the healthy controls), which may have potentially led to the misinterpretation of findings and mechanisms related to COVID-19. In addition, sample sizes for certain analyses, e.g., those exploring associations with certain drugs or vaccine subtypes, were low. Nonetheless, a key strength of our study is the overall large and diverse patient sample, which is especially important given the epidemiology of SLE including propensity in certain ethnic groups. In addition, the use of self-reported data allows for the inclusion of the patient perspective and minimises potential bias introduced by data collection by healthcare professionals, especially with regard to sensitive topics such as mental health; the latter is especially pertinent in the SLE population, where mental health conditions are highly prevalent.

## 5. Conclusions

Our study has provided overall reassuring data for the use of COVID-19 vaccines in people with SLE with regard to delayed AEs. While several minor AEs were reported with increased frequency in people with SLE compared to HCs, these were largely self-limiting and/or uncommon. However, it should not be overlooked that patients with SLE more frequently reported hospitalisations compared with healthy individuals and patients with non-rheumatic autoimmune conditions. Our data suggest there may be value in monitoring people with SLE following COVID-19 vaccination to help identify DAEs early and support patients in their care, especially those with autoimmune multimorbidity. Overall, our data contribute to the growing body of evidence that supports the uptake of COVID-19 vaccines in this vulnerable group of individuals. 

## Figures and Tables

**Table 1:** Sociodemographic and vaccination data for survey respondents.

Variable	Total, n [7203] (%) [100]	SLE, n [882] (%) [12.2]	rAIDs, n [3161] (%) [43.9]	nrAIDs, n [426] (%) [5.9]	HCs, n [2734] (%) [38.0]
**Age (median, IQR), years**	44 (34–56)	39 (31–50)	52 (41–62)	43 (34–53)	38 (30–49)
**Gender F:M**	5310:1799 3:1	826:46 18:1	2522:602 4.2:1	351:70 5:1	1611:1081 1.5:1
**Ethnicity n (%)**					
African American or of African origin (Black)	336 (4.7)	89 (10.1)	146 (4.6)	4 (0.9)	97 (3.5)
Asian	1632 (22.7)	263 (29.8)	602 (19.0)	57 (13.4)	710 (26.0)
Caucasian (White)	3039 (42.2)	274 (31.1)	1784 (56.4)	227 (53.3)	754 (27.6)
Do not wish to disclose	262 (3.6)	43 (4.9)	90 (2.8)	14 (3.3)	115 (4.2)
Hispanic	1200 (16.7)	114 (12.9)	283 (9.0)	83 (19.5)	720 (26.3)
Native American, Indigenous, Pacific Islander	53 (0.7)	12 (1.4)	17 (0.5)	4 (0.9)	20 (0.7)
Other	628 (8.7)	80 (9.1)	224 (7.1)	34 (8.0)	290 (10.6)

HCs: health controls; IQR: interquartile range; nrAID: non-rheumatic autoimmune disease; rAID: rheumatic autoimmune disease other than SLE; SLE: systemic lupus erythematosus.

**Table 2:** Effects of COVID-19 vaccination in patients with SLE versus HCs.

	SLE	HCs	Univariable		Multivariable	
	N 882 (%)	N 2734 (%)	OR (95% CI)	*p* Value	OR (95% CI)	*p* Value
**Minor AEs**	155 (17.6)	456 (16.7)		0.555		
**Injection site (arm) pain and soreness**	96 (10.9)	305 (11.2)		0.800		
Myalgia	73 (8.3)	164 (6.0)	**1.4 (1.1–1.9)**	**0.020**		0.079
Body ache	81 (9.2)	176 (6.4)	**1.5 (1.1–1.9)**	**0.007**		0.056
Joint pain	73 (8.3)	120 (4.4)	**1.9 (1.4–2.6)**	**<0.001**	**1.8 (1.3–2.6)**	**<0.001**
Fever	63 (7.1)	205 (7.5)		0.700		
Chills	52 (5.9)	127 (4.6)		0.137		0.183
Cough	18 (2.0)	34 (1.2)		0.084		
Difficulty in breathing or shortness of breath	24 (2.7)	33 (1.2)	**2.3 (1.3–3.9)**	**0.002**	**1.9 (1.1–3.4)**	**0.031**
Nausea/vomiting	39 (4.4)	36 (1.3)	**3.5 (2.2–5.5)**	**<0.001**	**3.0 (1.8–5.0)**	**<0.001**
Headache	71 (8.0)	142 (5.2)	**1.6 (1.2–2.1)**	**0.002**	**1.5 (1.1–2.1)**	**0.012**
Rash	23 (2.6)	21 (0.8)	**3.5 (1.9–6.3)**	**<0.001**	**3.4 (1.7–6.6)**	**<0.001**
Fatigue	92 (10.4)	136 (5.0)	**2.2 (1.7–2.9)**	**<0.001**	**2.0 (1.5–2.8)**	**<0.001**
Diarrhoea	21 (2.4)	33 (1.2)	**2.0 (1.1–3.5)**	**0.012**		0.066
Abdominal pain	14 (1.6)	16 (0.6)	**2.7 (1.3–5.6)**	**0.004**	**2.7 (1.2–5.9)**	**0.015**
High pulse rate or palpitations	19 (2.2)	45 (1.6)		0.320		
Rise in blood pressure	17 (1.9)	19 (0.7)	**2.8 (1.5–5.4)**	**0.001**	**2.7 (1.3–5.6)**	**0.009**
Fainting	4 (0.5)	9 (0.3)		0.592		
Dizziness	33 (3.7)	51 (1.9)	**2.0 (1.3–3.2)**	**0.001**	**2.2 (1.3–3.5)**	**0.002**
Chest pain	22 (2.5)	29 (1.1)	**2.4 (1.4–4.2)**	**0.002**	**2.3 (1.2–4.3)**	**0.009**
Swelling in the extremities	14 (1.6)	17 (0.6)	**2.6 (1.3–5.3)**	**0.007**	**2.4 (1.1–5.1)**	**0.027**
Weakness and tingling in the feet and legs	28 (3.2)	49 (1.8)	**1.8 (1.1–2.9)**	**0.013**		0.093
Pricking or pins and needles sensations in the hands and feet	20 (2.3)	28 (1.0)	**2.2 (1.3–4.0)**	**0.005**	**1.9 (1.0–3.7)**	**0.040**
Visual disturbances (e.g., loss of vision, blurring of vision)	20 (2.3)	21 (0.8)	**3.0 (1.6–5.6)**	**<0.001**	**3.5 (1.7–7.2)**	**0.001**
Bleeding/bruising on the body	15 (1.7)	9 (0.3)	**5.2 (2.3–12.0)**	**<0.001**	**3.9 (1.6–9.5)**	**0.003**
Petechial rash	8 (0.9)	5 (0.2)	**5.0 (1.6–15.3)**	**0.002**	**4.2 (1.1–15.5)**	**0.030**
**Major AEs**	118 (13.4)	224 (8.2)	**1.7 (1.4–2.2)**	**<0.001**	**1.6 (1.2–2.0)**	**0.001**
Anaphylaxis	8 (0.9)	19 (0.7)		0.525		
Marked difficulty in breathing	24 (2.7)	42 (1.5)	**1.8 (1.1–3.0)**	**0.022**	**2.0 (1.1–3.5)**	**0.020**
Throat closure	8 (0.9)	13 (0.5)		0.143		0.054
Severe rashes	22 (2.5)	29 (1.1)	**2.4 (1.4–4.2)**	**0.002**	**2.2 (1.2–4.1)**	**0.011**
**Hospitalisation**	51 (5.8)	57 (2.1)	**2.9 (2.0–4.2)**	**<0.001**	**2.2 (1.4–3.4)**	**<0.001**

Multivariable logistic regression analysis adjusted for age, sex, and ethnicity. AE: adverse event; CI: confidence interval; HC: healthy control; OR: odds ratio; SLE: systemic lupus erythematosus.

**Table 3:** Effects of COVID-19 vaccination in patients with SLE vs. rAIDs.

	SLE	rAIDs	Univariable		Multivariable	
	N 882 (%)	N 3161 (%)	OR (95% CI)	*p* Value	OR (95% CI)	*p* Value
**Minor AEs**	155 (17.6)	516 (16.3)		0.378		
**Injection site pain and/or soreness**	96 (10.9)	301 (9.5)		0.229		
Myalgia	73 (8.3)	223 (7.1)		0.218		
Body ache	81 (9.2)	245 (7.8)		0.167		0.682
Joint pain	73 (8.3)	242 (7.7)		0.543		
Fever	63 (7.1)	188 (5.9)		0.193		0.724
Chills	52 (5.9)	153 (4.8)		0.207		
Cough	18 (2.0)	49 (1.6)		0.313		
Difficulty in breathing or shortness of breath	24 (2.7)	60 (1.9)		0.130		0.763
Nausea/vomiting	39 (4.4)	72 (2.3)	**2.0 (1.3–3.0)**	**0.001**		0.076
Headache	71 (8.0)	213 (6.7)		0.178		0.977
Rash	23 (2.6)	78 (2.5)		0.814		
Fatigue	92 (10.4)	258 (8.2)	**1.3 (1.0–1.7)**	**0.034**		0.228
Diarrhoea	21 (2.4)	55 (1.7)		0.215		
Abdominal pain	14 (1.6)	43 (1.4)		0.613		
High pulse rate or palpitations	19 (2.2)	72 (2.3)		0.827		
Rise in blood pressure	17 (1.9)	39 (1.2)		0.119		0.249
Fainting	4 (0.5)	11 (0.3)		0.649		
Dizziness	33 (3.7)	109 (3.4)		0.676		
Chest pain	22 (2.5)	54 (1.7)		0.129		0.924
Swelling in the extremities	14 (1.6)	50 (1.6)		0.991		
Weakness and tingling in the feet and legs	28 (3.2)	78 (2.5)		0.245		
Pricking or pins and needles sensations in the hands and feet	20 (2.3)	68 (2.2)		0.834		
Visual disturbances (e.g., loss of vision, blurring of vision)	20 (2.3)	44 (1.4)		0.065		0.194
Bleeding/bruising on the body	15 (1.7)	29 (0.9)	**1.9 (1.0–3.5)**	**0.047**		0.146
Petechial rash	8 (0.9)	20 (0.6)		0.385		
**Major AEs**	118 (13.4)	342 (10.8)	**1.3 (1.0–1.6)**	**0.034**		0.610
Anaphylaxis	8 (0.9)	22 (0.7)		0.519		
Marked difficulty in breathing	24 (2.7)	51 (1.6)	**1.7 (1.0–2.8)**	**0.031**		0.354
Throat closure	8 (0.9)	23 (0.7)		0.589		
Severe rashes	22 (2.5)	40 (1.3)	**2.0 (1.2–3.4)**	**0.009**	**2.4 (1.3–4.2)**	**0.004**
**Hospitalisation**	51 (.5.8)	136 (4.3)		0.064		0.649

Multivariable logistic regression analysis adjusted for age, sex, and ethnicity. AE: adverse event; CI: confidence interval; OR: odds ratio; rAID: rheumatic autoimmune disease other than SLE; SLE: systemic lupus erythematosus.

**Table 4:** Effects of COVID-19 vaccination in patients with SLE vs. nrAIDs.

	SLE	nrAIDs	Univariable		Multivariable	
	N 882 (%)	N 426 (%)	OR (95% CI)	*p* Value	OR (95% CI)	*p* Value
**Minor AEs**	155 (17.6)	68 (16.0)		0.468		
**Injection site pain and/or soreness**	96 (10.9)	27 (6.3)	**1.8 (1.2–2.8)**	**0.008**	**1.8 (1.1–3.0)**	**0.014**
Myalgia	73 (8.3)	16 (3.8)	**2.3 (1.3–4.0)**	**0.002**	**2.0 (1.1–3.7)**	**0.018**
Body ache	81 (9.2)	17 (4.0)	**2.4 (1.4–4.2)**	**0.001**	**2.1 (1.2–3.7)**	**0.008**
Joint pain	73 (8.3)	15 (3.5)	**2.5 (1.4–4.3)**	**0.001**	**2.4 (1.3–4.3)**	**0.005**
Fever	63 (7.1)	15 (3.5)	**2.1 (1.2–3.7)**	**0.010**	**2.0 (1.1–3.7)**	**0.022**
Chills	52 (5.9)	13 (3.1)	**2.0 (1.1–3.7)**	**0.027**	**2.0 (1.0–3.9)**	**0.039**
Cough	18 (2.0)	6 (1.4)		0.425		
Difficulty in breathing or shortness of breath	24 (2.7)	8 (1.9)		0.355		
Nausea/vomiting	39 (4.4)	7 (1.6)	**2.8 (1.2–6.2)**	**0.011**	**3.0 (1.2–7.3)**	**0.017**
Headache	71 (8.0)	20 (4.7)	**1.8 (1.1–3.0)**	**0.025**	**1.8 (1.1–3.2)**	**0.033**
Rash	23 (2.6)	3 (0.7)	**3.8 (1.1–12.6)**	**0.021**		0.061
Fatigue	92 (10.4)	25 (5.9)	**1.9 (1.2–3.0)**	**0.007**	**2.0 (1.2–3.2)**	**0.007**
Diarrhoea	21 (2.4)	9 (2.1)		0.762		
Abdominal pain	14 (1.6)	5 (1.2)		0.558		
High pulse rate or palpitations	19 (2.2)	14 (3.3)		0.221		
Rise in blood pressure	17 (1.9)	7 (1.6)		0.720		
Fainting	4 (0.5)	0 (0.0)		0.164		0.999
Dizziness	33 (3.7)	9 (2.1)		0.118		0.151
Chest pain	22 (2.5)	6 (1.4)		0.204		
Swelling in the extremities	14 (1.6)	2 (0.5)		0.085		0.141
Weakness and tingling in the feet and legs	28 (3.2)	7 (1.6)		0.108		0.287
Pricking or pins and needles sensations in the hands and feet	20 (2.3)	10 (2.3)		0.928		
Visual disturbances (e.g., loss of vision, blurring of vision)	20 (2.3)	6 (1.4)		0.297		
Bleeding/bruising on the body	15 (1.7)	2 (0.5)		0.066		0.160
Petechial rash	8 (0.9)	1 (0.2)		0.168		0.202
**Major AEs**	118 (13.4)	44 (10.3)		0.117		0.280
Anaphylaxis	8 (0.9)	1 (0.2)		0.168		0.284
Marked difficulty in breathing	24 (2.7)	10 (2.3)		0.691		
Throat closure	8 (0.9)	3 (0.7)		0.707		
Severe rashes	22 (2.5)	3 (0.7)	**3.6 (1.1–12.1)**	**0.027**		0.056
**Hospitalisation**	51 (5.8)	9 (2.1)	**2.8 (1.4–5.8)**	**0.003**	**2.3 (1.1–4.9)**	**0.029**

Multivariable logistic regression analysis adjusted for age, sex, and ethnicity. AE: adverse event; CI: confidence interval; nrAID: non-rheumatic autoimmune disease; OR: odds ratio; SLE: systemic lupus erythematosus.

## Data Availability

The datasets generated and/or analysed during the current study are not publicly available but are available from the corresponding author upon reasonable request.

## Supplementary Materials

The following supporting information can be downloaded at: https://www.mdpi.com/article/10.3390/jcm12247542/s1, **Supplementary Table S1**. List of rheumatic and non-rheumatic autoimmune diseases. **Supplementary Table S2.** List of minor and major adverse events (AEs). **Supplementary Table S3.** Significant adverse events following the Oxford/AstraZeneca and Moderna vaccines. **Supplementary Table S4.** Adverse events in people with SLE receiving methotrexate (MTX), mycophenolate mofetil (MMF), and hydroxychloroquine (HCQ). COVID-19 Vaccination in Autoimmune Diseases-2 (COVAD-2) Study Group—Complete Author List and Affiliations.
